# Determination of Oxidative Stress Related Toxicity on Repeated Dermal Exposure of Hydroxyapatite Nanoparticles in Rats

**DOI:** 10.1155/2014/476942

**Published:** 2014-12-21

**Authors:** Mohanan Parayanthala Valappil, Syama Santhakumar, Sabareeswaran Arumugam

**Affiliations:** ^1^Toxicology Division, Biomedical Technology Wing, Sree Chitra Tirunal Institute for Medical Sciences and Technology, Thiruvananthapuram, Kerala 695 012, India; ^2^Histopathology Laboratory, Biomedical Technology Wing, Sree Chitra Tirunal Institute for Medical Sciences and Technology, Thiruvananthapuram, Kerala 695 012, India

## Abstract

Hydroxyapatite nanoparticles (HANPs) have numerous applications, such as substitute for bone grafting, bone fillers, bioceramic coating, and dental fillings. The toxicity of these nanomaterials is of growing concern despite their significant scientific interest and promising potential in many applications. In this study, an in-house synthesized, characterized HANP of size <50 nm was investigated for the dermal toxicity. A paste of HANPs was prepared in water and applied on the dorsal side of the rats for 28 days. At the end of 28 days, blood was subjected to haematological and biochemical analysis. Gross necropsy was conducted and major organs were collected for histopathological observations. Liver from the animals was evaluated for lipid peroxidation, reduced glutathione, and antioxidant enzymes activity. It was observed that none of the animals showed any abnormality during the experimental period. Gross examination of carcasses did not reveal any abnormality in the organs examined. The results also demonstrated that there was no significant fluctuation in the level of antioxidant defense mechanisms, lipid peroxidation, and haematological and biochemical parameters. There was no histopathological lesion observed in any of the organs. Hence, it can be concluded that the synthesized HANPs were nontoxic at cellular level, when exposed dermally to rats.

## 1. Introduction

Toxicity of nanomaterials refers to the interaction of nanomaterials with the biological systems and induction of toxic responses. This may depend on the physicochemical characteristics as well as route of exposure. Once inside the body, they get distributed to various organs or may remain in the same site and they could be structurally modified or metabolized. Advances in nanotechnology led to the exposure of humans to engineered nanomaterials and hence it became necessary to evaluate the potential human health effects before these materials are fully exploited. Nanoparticles are a diverse class of small-scale (<100 nm) substances with novel properties like small size, large surface area, particular shape, and surface activity. Nanomaterial toxicology is an important subdiscipline of nanotechnology, which deals with the possible toxic effects of nanomaterials [1]. It was reported that toxicity of nanoparticles depends on their size, composition, surface functionalization, and so forth. The major toxicological issue associated with the manufactured nanomaterials is that some of them are redox active; it can be transported across cell membranes and interact with subcellular organelles. As a consequence of all these properties, nanoparticles can have direct interaction with individual target cells, either with the external membrane or inside the cell at the site of action [2].

The nanomaterials are increasingly being used for commercial purposes such as fillers, opacifiers, catalysts, semiconductors, cosmetics, and microelectronics. In health care systems also, nanomaterials have gained much research interest particularly in imaging and targeted drug delivery systems, of which magnetic nanoparticles are particularly appealing and are widely used in biomedical applications such as contrast agents in magnetic resonance imaging (MRI) [3], tissue repairing [4], detoxification of biological fluids, hyperthermia [5], drug delivery [6], cell separation [7], and drug targeting [8]. Ferrite particles coated with biocompatible phases like hydroxyapatite are employed for hyperthermia treatment of cancer [9].

With respect to toxicology, particle size and surface area are the most important physicochemical characteristics that determine the toxicity of any material. With the smaller size of nanomaterial, it is possible to elicit a variety of cell-material interactions that could lead to toxicological effects. Because of their small size, they can pass the cellular barrier and can be distributed systemically from the site of injection. Upon inhalation, they can translocate out of the respiratory tract via different pathways and mechanisms. On ingestion, systemic uptake of nanomaterials via lymph can occur. From the blood circulation, they can be distributed throughout the organism, and they will be taken up by liver, spleen, bone marrow, heart, and other major organs [1, 8].

Cellular antioxidant defense system includes free radical scavengers such as glutathione (GSH), low-molecular-weight tripeptide as well as antioxidant enzymes (superoxide dismutase (SOD), glutathione reductase (GR), and glutathione peroxidase (GPX) [1]. Free radicals have an affinity to damage the DNA bases leading to their modifications. It has been shown that metal and metal oxide nanoparticles induce DNA damage and apoptosis through ROS generation and oxidative stress [10, 11]. Due to the high reactivity of ROS, most cellular components are likely to be targets of oxidative damage: lipid peroxidation, protein oxidation, GSH depletion, and DNA single strand breaks are all initiated by the excess of ROS. All of these events ultimately lead to cellular dysfunction and injury [12]. For this reason antioxidant enzymes are vital markers for oxidative stress induced in the body. Aerobic organisms possess antioxidant defense systems that deal with the removal of ROS. As long as there exists a balance between oxidative stress and antioxidant defense system, human body is maintained in an optimal health state.

Delivery of nanomaterials through skin has potential application in drug delivery because of its large surface area and is even challenging because skin acts as a barrier from the external environment. Depending on the duration of exposure, these materials can translocate from circulation to internal organs. Hence the potential for significant biological response at each of these sites requires investigation [13]. It was observed that TiO_2_ (10–50 nm) could penetrate through the skin and reaches stratum corneum and even the dermis following repeated application. This may be due to leaching of particles from the site of exposure and results in systemic adverse effects [10]. Mohanan et al. [14] reported that dextran coated ferrite nanoparticles failed to induce delayed hypersensitivity or did not significantly alter the level of lipid peroxidation, reduced glutathione, glutathione reductase, glutathione peroxidase, superoxide dismutase, or oxidative stress related DNA damage. Hence the present study was planned to assess the dermal toxicity and associated oxidative stress related cellular toxicities of an in-house synthesized HANPs in Wistar rats.

## 2. Materials and Methods

HANPs (SCTIMST, India), thiobarbituric acid (TBA), reduced glutathione (GSH), oxidized glutathione (GSSG), dithiobis-2-nitrobenzoic acid (DTNB) (all from Sigma, USA), disodium hydrogen phosphate (Na_2_HPO_4_), sodium dihydrogen phosphate (NaH_2_PO_4_), ethylene diamine tetraacetic acid (EDTA), diethylene triamine penta-acetic acid (DTPA), trichloroacetic acid (TCA), and physiological saline were used. All the chemicals and reagents used were of analytical grade.

Spectrophotometer (Shimadzu, Japan), laminar air flow (Mark Air Particulars, India), incubator shaker (New Brunswick Scientific, USA), spectrophotometer (Shimadzu 1601, Japan), biophotometer (Eppendorf, Germany), and steam sterilizer (Nat Steel, India) were also used.

### 2.1. Animal Husbandry and Experimental Animals

All animals were handled humanely, without pain or distress and with due care for their welfare. The care and management of the animals will comply with the regulations of the Committee for the Purpose of Control and Supervision of Experimental Animals (CPCSEA), Government of India. All animal experiments were carried out after prior approval from Institutional Animal Ethics Committee and in accordance with approved institutional protocol.

Healthy Wistar rats weighing 200–250 g were maintained in a 12 h light/dark cycle at a constant temperature of 22 ± 3°C with free access to standard pellet diet and water. Individual animals were identified with picric acid marks on rats. In addition to this, each animal cage was identified by labels having details such as experiment number, name, animal number(s), and date of experiment. All the animals were acclimatized for a period of 5 days before initiation of experiment.

### 2.2. Synthesis and Characterization of HANPs

HANPs were synthesized by wet chemical method, where calcium phosphate was precipitated from the aqueous solution of calcium nitrate tetrahydrate (Ca(NO_3_)_2_ *·*4H_2_O) and ammonium dihydrogen orthophosphate (NH_4_H_2_PO_4_) (RANKEM, India). Precipitation was carried out at a pH of 11 at 0°C for 2 h. After ageing for 24 h, the precipitate was washed in distilled water, freeze-dried, and calcined at 300°C. The calcined precipitate was ball-milled and sieved to collect particle of size below 50 nm.

The synthesized HANPs were physicochemically characterized using standard techniques. Transmission electron microscopy (TEM) was performed to obtain the particle size using TEM (H-600). The infrared spectrum of HANPs was compared with standard material using Nicolet Impact 410 FT-IR spectroscopy and X-ray diffraction (XRD) spectrum was recorded in a diffractometer (Siemens D5005) for phase purity. The zeta potential of the HANP in buffer and water was analyzed using a Malvern Zeta sizer. In order to establish the stability of HANPs in the cell suspension, the sample was freeze-dried (in cell suspension) and observed in EDS and scanning electron microscopy (SEM) analysis. The details of characterization of HANPs were reported by Geetha et al. [15].

### 2.3. Dermal Toxicity

Dermal toxicity is to provide information on the possible health hazards likely to rise from the repeated exposures by dermal route over a limited period of time usually up to 28 days. Different concentrations (25, 50, and 100 mg/kg) of HANPs were exposed dermally to all animals up to 6 hours per day on a basis of 7 days per week for 28 days. At the end of the observation period, blood was collected from orbital sinus and subjected to haematological (WBC, RBC, haemoglobin, hematocrit, clotting time, platelet count, MCV, MCH, and MCHC) and biochemical (glucose, cholesterol, triglycerides, total bilirubin, albumin, calcium, phosphorus, chloride, total protein, creatinine, urea, SGPT, SGOT, alkaline phosphatase, and GGT) parameters (OECD).

Experimental animals were sacrificed by cervical dislocation and gross necropsies were performed on all animals which include examination of the external surface of the body and internal organs. Major organs (heart, liver, lungs, spleen, and skin) were preserved in 10% buffered formalin and subjected to histopathological studies. The fixed tissues were processed in an automated tissue processor (Leica ASP 300) and sectioned (5 *μ*m thin sections) using a rotary microtome (RM2255) and stained with hematoxylin and eosin (H&E). Stained sections were examined under a light microscope (Axio Imager Z1, Carl Zeiss) for the evidence of tissue lesions (see Table 1).

Liver from the experimental animals was collected, homogenized, and subjected to total protein, lipid peroxidation, reduced glutathione, and antioxidant enzymes (glutathione reductase, glutathione peroxidase, and superoxide dismutase).

### 2.4. Preparation of Liver Homogenate

Liver from the experimental animals was collected, washed in normal saline, and immediately placed in ice bath. 10% of liver tissue homogenate was prepared in phosphate buffer (0.1 M, pH 7.4) using an ice-chilled glass homogenizing vessel in a rotor stator homogenizer at 900 rpm and centrifuged at 3500 rpm for 10 min at 4°C in a refrigerated centrifuge. The resultant supernatants were maintained in an ice bath until being used for the estimation of total protein, lipid peroxidation (LPO), reduced glutathione (GSH), glutathione reductase (GR), glutathione peroxidase (GPx), and superoxide dismutase (SOD) using standard protocols with slight modifications.

### 2.5. Total Protein

Total proteins in the liver homogenate were estimated by the method of Lowry et al. [16] using bovine serum albumin as standard.

### 2.6. Lipid Peroxidation (LPO)

The extent of LPO in the liver homogenate of rats exposed to HANPs was determined as the concentration of malondialdehyde (MDA) generated by the thiobarbituric acid reactive substances (TBARS), as described by Okado-Matsumoto and Fridovich  [17]. The amount of MDA formed was measured spectrophotometrically at 532 nm.

### 2.7. Reduced Glutathione

The level of GSH in the liver homogenate was determined by the method of Moron et al. [18], with slight modifications in which Ellman's reagent or DTNB (5,5′-dithiobis-(2-nitrobenzoic acid)) reacts with GSH to form a spectrophotometrically detectable product at 412 nm. The change in absorbance at 412 nm is a linear function of the GSH concentration in the reaction mixture and is based on the reaction of GSH with DTNB to give a compound that is absorbed at 412 nm. The amount of GSH was expressed as nmol/mg protein.

### 2.8. Antioxidant Enzymes

GR activity in liver homogenate of rats exposed to HANPs was determined by measuring the reduction of GSSG in the presence of NADPH as described by Mize and Langdon [19]. Briefly, this assay measures the rate of NADPH oxidation to NADP+, which is accompanied by a decrease in absorbance at 340 nm, which can be monitored spectrophotometrically. Thus, one GR unit is defined as the reduction of one *μ*M of GSSG per minute at 25°C and pH 7.6.

The activity of GPX from the liver homogenate of rats exposed to HANPs was assayed by the method described by Rotruck  [20]. The remaining GSH after the enzyme catalyzed reaction was complexed with DTNB, which is absorbed at a maximum wavelength of 412 nm. Enzyme activity was expressed as *μ*g of GSH consumed/min/mg protein.

SOD in the liver homogenate of rats was done using modified pyrogallol autooxidation method spectrophotometrically measured at 420 nm [21].

All measurements were carried out using UV Spectrophotometer-1601, Shimadzu, Japan.

### 2.9. Statistical Analysis

All values are expressed as mean ± SD. Statistical significance between the control and experimental values was compared by Student's *t*-test. For all comparisons, *P* < 0.05 was considered significant. 

## 3. Results

### 3.1. Synthesis and Characterization of HANPs

**Figure 1:**
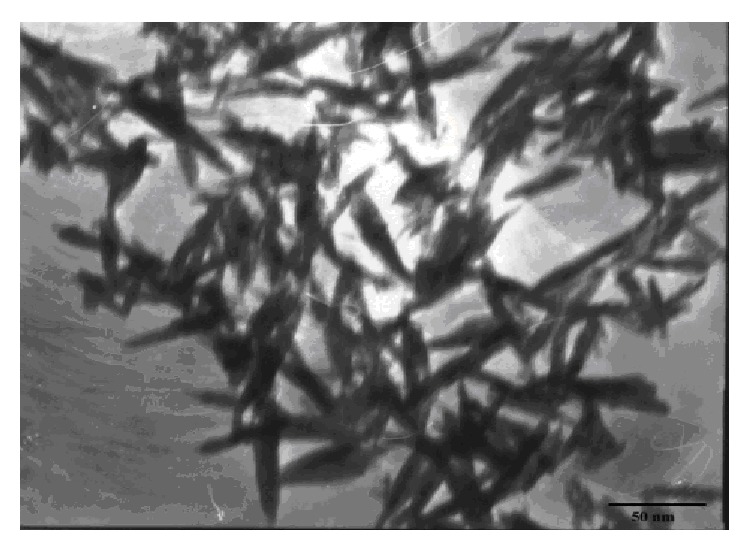
TEM image of an in-house synthesized HANP.

HANPs were synthesized by wet chemical method. These particles were characterized by transmission electron microscopy, X-ray diffraction analysis, Fourier transform infrared spectral analysis, zeta potential measurements, and SEM/EDS ( ). The transmission electron microscopic data indicated in Figure 1 suggested that the size of synthesized HANPs was found to be below 50 nm.

### 3.2. Dermal Toxicity

Different concentrations (25, 50, and 100 mg/kg) of HANPs (with control animals) were exposed to Wister rats on dermal route for a period of 28 days continuously. The general physical conditions of the experimental animals were normal. The increase in body weight and feed intake was normal and none of the animals showed any abnormality or behavioural changes during the experimental period.

The blood collected from the experimental animals was subjected to analyze the haematological (WBC, RBC, haemoglobin, hematocrit, clotting time, platelet count, MCV, MCH, and MCHC) and biochemical (glucose, cholesterol, triglycerides, total bilirubin, albumin, calcium, phosphorus, chloride, total protein, creatinine, urea, SGPT, SGOT, alkaline phosphatase, and GGT) parameters. The results of the haematological parameters were mentioned in Table 2 and it was found that the haematological values of animals exposed to different concentrations of HANPs were well comparable with control values, except for a slight reduction in haemoglobin, platelets, and MCH which is under normal range. Similarly there were no alternations observed in the biochemical values of treated and control animals. All the biochemical values are well under normal range except for one incidence in chlorides, total proteins, and alkaline phosphatase (Table 3).

Gross examination of carcasses of male and female control and treated groups animals did not reveal any gross abnormality in the major organs examined, such as heart, liver, lungs, spleen, and skin. Skin revealed focal pale hairless area observed in the control group animals. Similarly, skin revealed focal pale hairless brownish stained area observed in the dorsum of treated animals.

**Figure 2:**
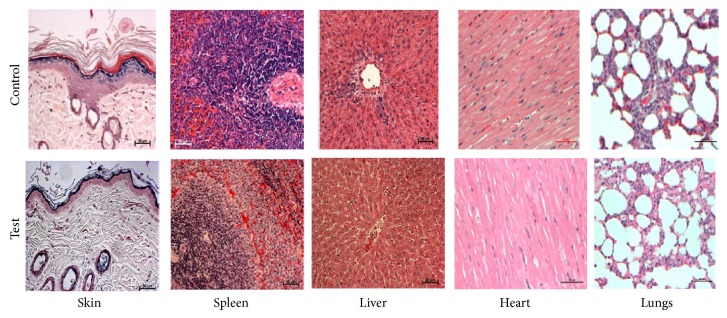
Photomicrographs of histopathology of skin, spleen, liver, heart, and lungs (test and control).

Histopathological examinations of the skin of control group rats revealed hyperkeratosis in all cases; four cases of spinous layer with multiple basophilic pigment layers and five cases of intracytoplasmic vacuolation and edema in basal cells were observed. In dermis, mild edema and mild infiltration were noted in all cases. Five cases revealed focal area of hypotrichosis. Sebaceous gland appeared normal in all cases (Figure 2).

Skin of treated group rats revealed hyperkeratosis, spinous layer with multiple basophilic pigment layers, and intracytoplasmic vacuolation and edema in basal cells. In dermis, mild edema and mild infiltration of mononuclear inflammatory cells (histiocytes and lymphocytes) were noted in all cases. All cases revealed focal area of hypotrichosis. Sebaceous glands appeared normal in all cases. Seven cases of bronchial associated lymphoid tissue proliferation were noted in the lungs. All other organs examined did not reveal any abnormality in both groups (Figure 2).

### 3.3. Lipid Peroxidation

**Figure 3:**
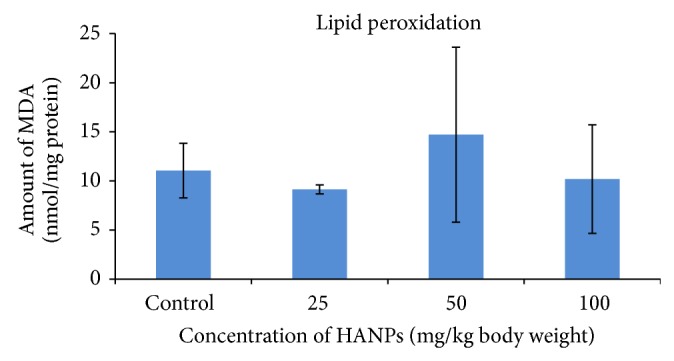
Level of malondialdehyde in the liver of rat exposed to HANPs (mean ± SD, *n* = 5).

Figure 3 defines the results of the LPO production. LPO was slightly reduced in the 25 mg/kg (9.13 ± 0.46 nmoles/mg protein) and 100 mg/kg (10.19 ± 5.53 nmoles/mg protein) HANPs exposed groups. However there was an increase in LPO observed in the 50 mg/kg HANPS exposed group (11.05128 ± 2.79 nmoles/mg protein), when compared to control (nmoles/mg protein).

### 3.4. Reduced Glutathione

**Figure 4:**
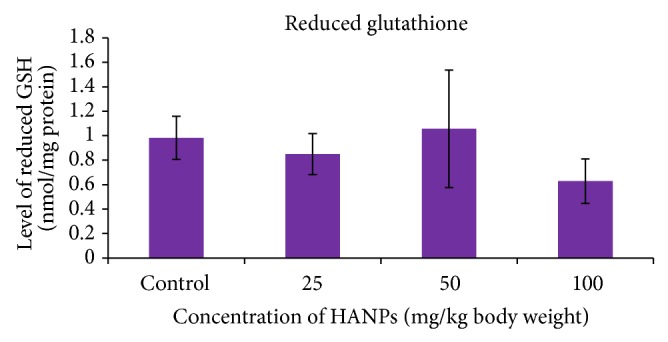
Level of GSH in the liver of rat exposed to HANPs (mean ± SD, *n* = 5).

The results of the GSH are shown in Figure 4. It was found that a decrease in the level of reduced glutathione was observed in the liver homogenates of rats exposed to HANPs dermally. The level of GSH was found to be reduced in 25 mg/kg (0.85 ± 0.17 nmol/mg) and 100 mg/kg (0.63 ± 0.18 nmol/mg) HANPs exposed groups, respectively; however, GSH was slightly increased in the 50 mg/kg exposed group (1.06 ± 0.48 nmol/mg), when compared to control (0.98 ± 0.18 nmol/mg), and was not significant.

### 3.5. Antioxidant Enzymes

**Figure 5:**
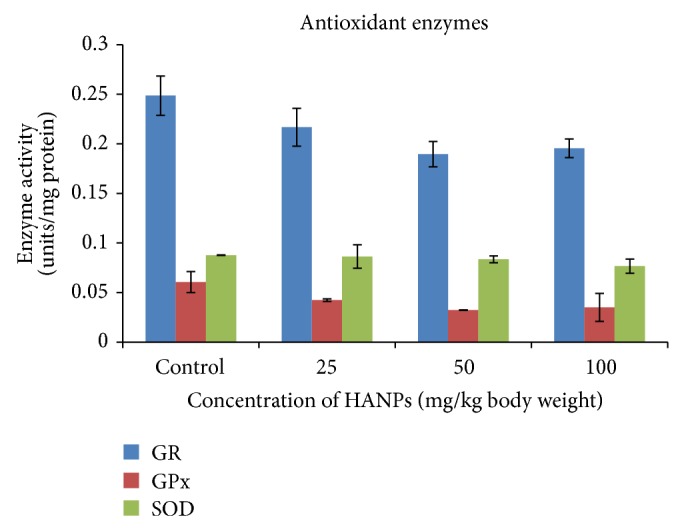
Activity of antioxidant enzymes in the liver of rat exposed to HANPs (mean ± SD, *n* = 5).

The result of the antioxidant enzymes was indicated in Figure 5. The level of GR was slightly reduced in 25 mg/kg (0.22 ± 0.02 units/mg protein), 50 mg/kg (0.19 ± 0.0102 units/mg protein), and 100 mg/kg dose (0.19 ± 0.0102 units/mg protein) HANPs exposed groups, when compared to control group (0.25 ± 0.0202 units/mg protein), and was not significant.

A dose dependant decrease in the activity of GPx was observed in HANPs exposed animals with the values of 0.042 ± 0.001 for 25 mg/kg, 0.032 ± 0.001 for 50 mg/kg, and 0.035 ± 0.014 for 100 mg/kg. These values were not significant when compared to control values.

Likewise, a slight decrease in the SOD activity was observed in 25, 50, and 100 mg/kg body weight of HANPs exposed groups (0.086 ± 0.011, 0.084 ± 0.003, and 0.077 ± 0.007), when compared to control group (0.088 ± 0.001), and was not significant.

## 4. Discussion

Tan et al. [10] reported that TiO_2_ (10–50 nm) could penetrate through the skin and reaches stratum corneum and even the dermis following repeated application. This may be due to leaching of particles from the site of exposure and results in systemic adverse effects. Similarly it was showed that the delivery of nanomaterials through skin has potential application in drug delivery because of its large surface area and is even challenging because skin acts as a barrier from the external environment. Depending on the duration of exposure, these materials can translocate from circulation to internal organs. Hence the potential for significant biological response at each of these sites requires investigation [13]. Based on these findings, it was planned to assess the dermal toxicity and associated oxidative stress related cellular toxicities of an in-house synthesized HANP in Wistar rats.

The in-house synthesized HANPs were characterized by transmission electron microscopy, X-ray diffraction analysis, Fourier transform infrared spectral analysis, zeta potential measurements, and SEM/EDS. The transmission electron microscopic data indicated that the size of synthesized HANP particle was below 50 nm [22].

Dermal exposure of HANPs in Wistar rats for 28 days continuously showed that the general physical conditions of the experimental animals were normal during the experimental period. It was found that there was no alternation in the haematological values. Biochemical parameters did not show any variations, indicating the normal functioning of liver, when different concentrations of HANPs were exposed to rats for 28 days. The fluctuations observed in few of the haematological and biochemical parameters were under normal range.

The results of the gross examination suggested that the carcasses of control and HANPs exposed rats (male and female) did not reveal any major abnormality in the organs examined. It was observed that the histopathological (heart, liver, lungs, spleen, and skin) examination did not reveal any major abnormalities in the sections examined. This suggests that the lesions observed in the histopathological findings of both HANPs treated and control animals may not be related to the dermal exposure of HANPs. A similar response was observed in our previous study where dextran coated ferrite nanomaterials were exposed dermally to rats for 28 days [14].

The result of the LPO studies indicated that there was a slight alternation in the medium dose group (not significant) when compared to control values. It was found that the HANPs did not induce any changes in lipid peroxidation or free radical generation at a dose of 100 mg/kg body weight. It was well established that lipid oxidation is a major harmful consequence of ROS formation [23, 24] as it reflects irreversible oxidative changes of membranes. Unsaturated lipids in liver tissue are very susceptible to peroxidation when they are exposed to ROS. Malondialdehyde (MDA) is the principal and the most studied product of polyunsaturated fatty acid peroxidation [25]. The concentration of MDA in biological materials has been widely utilized as an indicator of oxidative damage to unsaturated lipid. The level of MDA is determined by its derivatization with TBA. Measurement of MDA, the byproduct of LPO, provides an exact and well established index of oxidative damage since it is very reactive and takes part in cross-linking with biomolecules [26].

In the present study on exposure of different concentrations of HANPs to rat did not alter the GSH levels. This is in support of our earlier studies stating that the dextran coated ferrite nanomaterials did not induce any alternations in the GSH levels [14]. It was also reported [27] that after administration of nanoparticles GSH can act as a conjugating agent in their metabolism. When these nanoparticles induce oxidative stress by generating H_2_O_2_ or hydroperoxides, GSH can also be oxidized in a reaction catalyzed by GSH-Px. It was also reported that depletion of GSH in tissues leads to impairment of the cellular defense against ROS and may result in peroxidative injury [27, 28]. GSH plays a vital role in the protection of cells against oxidative stress and acts as an important aqueous-phase nonenzymatic antioxidant and an essential cofactor for antioxidant enzymes taking part in cellular redox reactions.

It is well established that the glutathione peroxidase functions in the scavenging and inactivation of hydrogen and lipid peroxides, thereby protecting the body against oxidative stress. The biochemical function of glutathione peroxidase is to reduce lipid hydroperoxides to their corresponding alcohols and to reduce free hydrogen peroxide to water [29]. Flohe [30] reported that GPx, GR, and SOD protect cells against ROS. GR and GPx are the two most important enzymes in the GSH-GSSG cycle and may be activated by increased hydrogen and/or lipid peroxide production. GPX reduces the lipid hydroperoxides to hydroxylated lipid derivatives. Hydrogen peroxide is rapidly converted into the toxic hydroxyl radical, which represents the main ROS product responsible for lipid peroxidation. Antioxidant activity of GSH-Px involves neutralization of H_2_O_2_, reduction of lipid hydroperoxidases, and maintenance of normal membrane permeability [31].

SOD is considered as the first line of defense against the deleterious effects of oxygen radicals in the cells and it scavenges ROS by catalyzing the dismutation of superoxide to H_2_O_2_ [21]. It is an enzyme found in all living cells, which speeds up certain chemical reactions in the body. This enzyme catalyzes the dismutation of superoxide into oxygen and hydrogen peroxide. The superoxide can cause mutations in DNA or attack enzymes that synthesise amino acids and other essential molecules. Thus, they are important antioxidant defence mechanisms of all cells exposed to oxygen. It helps the breakdown of potentially harmful oxygen molecules in cells, which might prevent damage to tissues [32]. In the present study it was observed that there was a dose dependant reduction in the activity of antioxidant enzymes such as GR and GPx when compared to control values and it is not significant. However there was no change in the activities of SOD at any concentration of HANPs.

## 5. Conclusion

Based on the results of the study it can be concluded that the HANPs were nontoxic when exposed dermally. There was no significant fluctuation in the level of lipid peroxidation, reduced glutathione, antioxidant defense mechanism, or histopathological lesions. Hence, it can be concluded that the in-house synthesized HANPs were found to be nontoxic at cellular level and dermally to rats under laboratory conditions simulated. The above results will be complimentary to co-relate with transcription, translational levels of corresponding genes, and ADMET assays to confirm its prolonged effects.

## Figures and Tables

**Table 1:** Experimental design.

Group	Dose	Number of rats		Exposure route	Days of exposure
		Male	Female		
1	Control	5	5	Dermal	28
2	HANPs (25 mg/kg)	5	5	Dermal	28
3	HANPs (50 mg/kg)	5	5	Dermal	28
4	HANPs (100 mg/kg)	5	5	Dermal	28

HANPs: hydroxyapatite nanoparticles.

**Table 2:** Haematological values of rats when exposed to HANPs (mean ± SD, *n* = 5).

Parameters	Control	HANPs		
		(25 mg/kg)	(50 mg/kg)	(100 mg/kg)
WBC	5.64 ± 2.20	6.25 ± 1.51	5.85 ± 1.46	7.62 ± 2.22
RBC	6.09 ± 0.39	6.35 ± 0.48	6.14 ± 0.63	6.02 ± 0.63
Hb	15.77 ± 0.81	15.95 ± 0.92	15.46 ± 0.10	14.66 ± 0.91^*^
Hct	33.03 ± 1.82	34.58 ± 2.83	33.31 ± 3.18	32.12 ± 3.20
PLT	705.0 ± 106.66	702.7 ± 54.49	690.1 ± 85.74	600.9 ± 71.45^*^
MCV	54.5 ± 1.35	54.5 ± 1.08	54.4 ± 1.51	53.5 ± 0.71
MCH	25.91 ± 0.92	25.14 ± 0.76	25.3 ± 1.46	24.52 ± 1.33^*^
MCHC	47.75^*^ ± 1.39	46.2 ± 1.57	46.55 ± 2.17	45.89 ± 2.42

^*^Statistically significant *P* < 0.05; HANPs: hydroxyapatite nanoparticles.

**Table 3:** Biochemical values of rats when exposed to HANPs (mean ± SD, *n* = 5).

Parameters	Control	HANPs		
		(25 mg/kg)	(50 mg/kg)	(100 mg/kg)
Glucose	110.8 ± 35.47	88.6 ± 13.81	91.6 ± 15.78	91.2 ± 14.46
Cholesterol	60.2 ± 6.06	59.4 ± 4.56	63.0 ± 5.48	57.6 ± 5.73
Triglycerides	106.0 ± 18.37	122.0 ± 0.65	123.0 ± 0.65	112.6 ± 0.62
Bilirubin	0.18 ± 0.10	0.13 ± 0.04	0.09 ± 0.01	0.12 ± 0.044
Albumin	4.08 ± 0.32	4.36 ± 0.27	4.40 ± 0.25	4.14 ± 0.25
Calcium	10.68 ± 0.50	10.92 ± 0.38	10.68 ± 0.24	10.46 ± 0.47
Phosphate	9.05 ± 2.23	8.13 ± 1.71	7.94 ± 1.94	8.86 ± 1.83
Chlorides	108.6 ± 2.43	106.5 ± 1.65	104.1 ± 0.99^*^	109.5 ± 1.65
Total proteins	7.56 ± 0.27	7.54 ± 0.21	7.54 ± 0.41	7.52 ± 0.51
Creatinine	0.95 ± 0.09	0.86 ± 0.06	0.87 ± 0.07	0.89 ± 0.08
Urea	37.1 ± 2.62	38.6 ± 2.93	37.8 ± 3.30	38.4 ± 3.30
SGPT	132.2 ± 28.54	151.9 ± 34.61	107.5 ± 13.26	113.1 ± 21.82
SGOT	188.7 ± 79.28	123.2 ± 20.65	127.7 ± 8.62	153.0 ± 36.23
ALP	170.8 ± 24.01	195.4 ± 28.11	206.8 ± 38.52	204.6 ± 19.28^*^
GGT	8.28 ± 9.11	2.52 ± 1.74	4.34 ± 7.16	1.38 ± 0.29

^*^Statistically significant *P* < 0.05; HANPs: hydroxyapatite nanoparticles.
