# Corrigendum to “Determination of Oxidative Stress Related Toxicity on Repeated Dermal Exposure of Hydroxyapatite Nanoparticles in Rats”

**DOI:** 10.1155/2015/320547

**Published:** 2015-04-16

**Authors:** Parayanthala Valappil Mohanan, Santhakumar Syama, Arumugam Sabareeswaran

**Affiliations:** ^1^Toxicology Division, Biomedical Technology Wing, Sree Chitra Tirunal Institute for Medical Sciences and Technology, Thiruvananthapuram, Kerala 695 012, India; ^2^Histopathology Laboratory, Biomedical Technology Wing, Sree Chitra Tirunal Institute for Medical Sciences and Technology, Thiruvananthapuram, Kerala 695 012, India

In the paper titled “Determination of Oxidative Stress Related Toxicity on Repeated Dermal Exposure of Hydroxyapatite Nanoparticles in Rats,” the first and last names of all the authors were reversed. The correct names in order (first name, last name) have been shown above.

